# Teilhabe in Sportvereinen fördern: Sport- und bewegungsorientierte Soziale Arbeit bei Menschen mit Beeinträchtigung

**DOI:** 10.1007/s00103-026-04317-4

**Published:** 2026-10-08

**Authors:** Sina Eghbalpour, Pauline Reifferscheidt

**Affiliations:** Abteilung Aachen, Katholische Hochschule Nordrhein-Westfalen, Robert-Schuman-Str. 25, 52066 Aachen, Deutschland

**Keywords:** Inklusive Bewegungsförderung, Menschen mit Behinderungen, Sport und Gesundheit, Soziale Teilhabe, Sport- und bewegungsorientierte Soziale Arbeit, Inclusive physical activity, Children and adolescents with disabilities, Health promotion, Social participation, Sport- and movement-oriented social work

## Abstract

Körperliche Aktivität ist eine wichtige Ressource für Gesundheit, Entwicklung und soziale Teilhabe von Kindern und Jugendlichen. Insbesondere für Menschen mit Beeinträchtigung kann Sport motorische Fähigkeiten, psychosoziales Wohlbefinden, Selbstwirksamkeit und gesellschaftliche Teilhabe fördern sowie zur Prävention chronischer Erkrankungen und Adipositas beitragen. Dennoch bestehen weiterhin erhebliche Barrieren beim Zugang zu inklusiven Sport- und Bewegungsangeboten.

Vor dem Hintergrund der UN-Behindertenrechtskonvention, insbesondere Artikel 30, beleuchtet der Beitrag praxisorientierte Ansätze der Bewegungsförderung für Menschen mit Beeinträchtigung. Auf Grundlage aktueller Forschung werden bestehende Diskrepanzen zwischen dem hohen Interesse an sportlicher Teilhabe und der tatsächlichen Nutzung bzw. dem Angebot organisierter Sportangebote aufgezeigt. Im Fokus steht das Potenzial der sport- und bewegungsorientierten sozialen Arbeit zur Förderung einer teilhabeorientierten Gesundheitsförderung.

Der Beitrag verdeutlicht, dass inklusive Bewegungsförderung barrierefreie Strukturen, qualifizierte Fachkräfte sowie eine enge Zusammenarbeit zwischen Sportvereinen, Schulen, Kinder- und Jugendhilfe, Familien, Assistenzdiensten und kommunalen Akteur*innen erfordert.

## Abstract (en)

Physical activity is an important resource for health, development and social participation in childhood and adolescence. For children and adolescents with disabilities, sport and movement can support motor development, psychosocial well-being, self-efficacy, and social inclusion, while also contributing to the prevention of chronic diseases and obesity. However, access to inclusive physical activity opportunities remains limited. Barriers include inaccessible facilities, insufficient transport and assistance services, a lack of low-threshold offers, and uncertainties among professionals in dealing with heterogeneous groups.

Against the background of the United Nations Convention on the Rights of Persons with Disabilities, particularly Article 30 on participation in recreation, leisure, and sport, this article discusses practice-oriented approaches to physical activity promotion for children and adolescents with disabilities. Drawing on findings on participation in organized sport, the article highlights a persistent gap between interest in sport participation and actual access to inclusive sport structures.

The article focuses on the potential of sport- and movement-oriented social work to strengthen participation-oriented health promotion. Particular attention is given to cooperation between sport clubs, schools, child and youth services, families, assistance services, and local communities. The article argues that inclusive physical activity promotion requires not only individual support, but also accessible structures, qualified professionals, and settings that take the everyday lives, preferences, and participation rights seriously.

## Einleitung

Besonders der organisierte Sport bietet vielfältige Möglichkeiten, die Teilhabe von Menschen mit Beeinträchtigung (MmB) am gesellschaftlichen Leben und die Förderung des allgemeinen Gesundheitszustandes positiv zu beeinflussen. Daneben wirkt sich die präventive und rehabilitative Funktion positiv auf Lebensqualität, sensomotorische Funktionen und psychosoziale Aspekte von Menschen mit Beeinträchtigung aus [1].

Welche Voraussetzungen müssen erfüllt sein, damit Sport tatsächlich inklusiv wird? Mehr als 18 Jahre nach Inkrafttreten der UN-Behindertenrechtskonvention (UN-BRK) ist der Anspruch auf gleichberechtigte Teilhabe von Menschen mit Beeinträchtigung im Sport noch immer nicht eingelöst. Die Konvention markierte einen Paradigmenwechsel vom Fürsorgeprinzip hin zu einem Menschenrechtsansatz, der die gleichberechtigte Teilhabe und Selbstbestimmung von Menschen mit Beeinträchtigung in allen Lebensbereichen einfordert, auch im Bereich der Bewegungsförderung – dem Sport.

Dennoch besteht eine deutliche Diskrepanz zwischen Anspruch und Praxis: Der Landessportbund Nordrhein-Westfalen [2] verweist darauf, dass trotz hoher Teilhabemotivation die Beteiligung von Menschen mit Beeinträchtigung in Sportvereinen gering ist. Strukturelle Barrieren wie fehlende Barrierefreiheit, unzureichende Informationen sowie mangelnde Unterstützung in den Bereichen Mobilität und Assistenz erschweren den Zugang [3].

Der Beitrag beleuchtet zentrale Ansatzpunkte für mehr Inklusion im Sport und untersucht, welche Bedeutung sport- und bewegungsorientierte Soziale Arbeit für die Förderung von Teilhabeprozessen in Sportvereinen hat. Im Fokus stehen dabei die Qualifizierung von Übungsleitungen, der Ausbau niedrigschwelliger Unterstützungsangebote sowie eine menschenrechtsorientierte Haltung im Umgang mit Menschen mit Beeinträchtigung. Inklusion im Sport erfordert ein koordiniertes Zusammenwirken von Sportvereinen, Sozialer Arbeit und Politik, um Bewegungsförderung und gesellschaftliche Teilhabe nachhaltig zu ermöglichen.

## Ausgangslage: Inklusion im Spannungsfeld sportpolitischer und rechtlicher Umsetzung

In den Debatten um die Begrifflichkeiten von Inklusion, Teilhabe und Partizipation von Menschen mit Beeinträchtigung wird der stark politisch konnotierte Begriff der Inklusion als wesentliche Grundlage für die insgesamt 50 Artikel der UN-Behindertenrechtskonvention genutzt. Demnach versteht sich Inklusion als sozialpolitischer Grundbegriff, welcher maßgeblich darauf abzielt, Teilhabemöglichkeiten für Menschen mit Beeinträchtigung in der Gesellschaft zu verbessern und Exklusion, im Sinne des sozialen Ausschlusses, zu verhindern. Ferner bezieht sich der Begriff auf grundlegende soziale Fragen der Konstruktion und der Umsetzung von Teilhabe und Vielfalt in unserer Gesellschaft, verbunden mit der Vision, Chancengerechtigkeit herzustellen und die gesellschaftliche Herausforderung von Verschiedenheit als Gemeinschaft zu bewältigen [4].

Besonders im Zuge der politischen Debatten um Teilhabe und Inklusion konnte die 2006 ratifizierte UN-Behindertenrechtskonvention einen neuen fundamentalen Grundstein für die Verbesserung der Teilhabe von MmB weltweit legen. Folgerichtig wird mit dem Inkrafttreten der UN-Behindertenkonvention 2009 Inklusion erstmals als ein Menschenrecht festgeschrieben. Damit trägt die Konvention zur Etablierung eines neuen Modells von Behinderung bei: des menschenrechtlichen Modells [5]. Degener [5] verweist eindrücklich darauf, dass bei der Entwicklung der einzelnen Artikel der UN-BRK keine neue Spezialkonvention entstehen sollte, vielmehr eine Erweiterung der bereits anerkannten Menschenrechte mit dem Bezug zu den Bedarfen von Menschen mit Beeinträchtigung. Auf Grundlage dieses Paradigmenwechsels wird Behinderung nicht länger als individuelles Schicksal betrachtet, sondern als Herausforderung und Aufgabe für einen sozialen und kulturellen Wandel einer ganzen Gesellschaft. Entsprechend dem Wunsch- und Wahlrecht werden passive Hilfeempfänger*innen zu aktiv beteiligten Personen in der Gesellschaft und somit auch im Sport.

Die UN-BRK fordert explizit in Artikel 30, die gleichberechtigte Teilhabe an Erholungs‑, Freizeit- und Sportaktivitäten zu ermöglichen. Besonders die in Artikel 30 benannten Abschnitte a–c rücken hier in den Vordergrund: die Ermutigung von MmB, so umfassend wie möglich an sportlichen Aktivitäten teilzunehmen, die Sicherstellung von Zugangsmöglichkeiten zu inklusiven Sportangeboten, Trainingszeiten, sportspezifischen Materialien sowie barrierefreien Sportstätten. Trotz der seit über 18 Jahren ratifizierten UN-BRK und bundesweiten Bemühungen ist eine gelungene und selbstverständliche Teilhabe am Sportsystem allerdings für viele MmB nach wie vor erheblich erschwert. Von den in Artikel 30 formulierten Rechtsansprüchen in Bezug auf gleichberechtigte Teilhabe an Erholungs‑, Freizeit- und Sportaktivitäten ist die praxisnahe Umsetzung leider noch weit entfernt [6–8].

Aktuelle Entwicklungen [9] verdeutlichen zudem, dass die Umsetzung der UN-Behindertenrechtskonvention in großen Teilen gefährdet ist. Ein vom Der Paritätische Gesamtverband veröffentlichtes Arbeitspapier von Bund, Ländern und kommunalen Spitzenverbänden weist auf weitreichende Kürzungsvorschläge im Bereich der Eingliederungshilfe hin. Diese betreffen insbesondere Leistungen zur sozialen Teilhabe und könnten bestehende Individualansprüche sowie das Wunsch- und Wahlrecht von Menschen mit Beeinträchtigung erheblich einschränken. Laut dieses aktuell vorliegenden Arbeitspapiers sind auch zentrale Voraussetzungen für eine gleichberechtigte Teilhabe im Sport betroffen. Insbesondere die Finanzierung von Assistenzleistungen und Mobilitätsangebote, die für viele Menschen mit Beeinträchtigung eine grundlegende Voraussetzung für die Teilnahme an Sport- und Bewegungsangeboten darstellen, geraten dadurch unter Druck. Einschränkungen in diesen Bereichen wirken sich unmittelbar auf die Möglichkeiten der Bewegungsförderung und damit auch auf bewegungsbezogene gesundheitliche Chancen aus. Vor diesem Hintergrund wird deutlich, dass die Frage der Inklusion und Teilhabe im Sport nicht allein eine Frage konzeptionellen Weiterentwicklung ist, sondern von politischen Prioritätensetzungen abhängt [9].

### Bedeutung des inklusiven Sports für Teilhabeprozesse

Bewegungsförderung im Kontext von Menschen mit Beeinträchtigung geht weit über den rein gesundheitlichen Aspekt hinaus. Sie stellt eine wichtige Form der sozialen Teilhabe dar und ermöglicht es, aktiv am gesellschaftlichen Leben mitzuwirken. Bewegung und Sport wirken dabei stets auf mehreren Ebenen gleichzeitig und entfalten dadurch eine ganzheitliche Wirkung. Auf körperlicher Ebene trägt Sport zur Verbesserung von Ausdauer, Koordination, Kondition und Entspannungsfähigkeit bei. Gleichzeitig stärkt er das Körperbewusstsein und unterstützt den Aufbau sowie die Weiterentwicklung motorischer Fähigkeiten. Ebenso bedeutsam sind die geistig-emotionalen Effekte: Durch Bewegung können Kreativität und Selbstvertrauen gefördert werden, während Erfolgserlebnisse das persönliche Wohlbefinden stärken und neue Motivation schaffen. Darüber hinaus werden auch kognitive und taktische Fähigkeiten geschult, die das eigenständige Handeln unterstützen [10]. Sport wirkt zudem motivationsfördernd und bietet insbesondere für Kinder und Jugendliche einen wichtigen Ausgleich zum schulischen Alltag. Von besonderer Bedeutung sind die sozialen Lernprozesse, die im gemeinsamen Sporttreiben entstehen. Mannschaftssport fördert Rücksichtnahme, gegenseitiges Verständnis und die Fähigkeit, Fairness aktiv zu leben. Gleichzeitig lernen die Sportler*innen, mit Erfolgen und Niederlagen umzugehen, Unterstützung zu geben und anzunehmen sowie Konflikte konstruktiv zu bewältigen. Gerade im Kontext der Arbeit mit Jugendlichen mit nicht sichtbaren Beeinträchtigungen sind regelgebundene und erlebnisorientierte Sportangebote zentral, um soziale Kompetenzen gezielt zu fördern. Ergänzend kommt wertschätzendem Feedback eine wichtige Rolle zu, da Lob und Anerkennung maßgeblich zur Stärkung des Selbstbewusstseins und zur Persönlichkeitsentwicklung beitragen [11].

Unkonventionelle und nicht leistungsorientierte Sportangebote im Verein bieten die Chance, ein Gemeinschaftsgefühl im Sportverein zu erleben, persönliche Grenzen auszutesten, Entwicklungspotenziale zu steigern und das Selbstwertgefühl sowie die Selbstwirksamkeit zu verbessern. Besonders inklusive Sportangebote können dazu beitragen, die Beeinträchtigung in den Hintergrund treten zu lassen und die Freude an der Bewegung in den Vordergrund zu stellen. Zudem kann der Sport nicht nur die mentale und physische Gesundheit fördern, sondern einen Beitrag dazu leisten, der gesellschaftlichen Vereinsamung präventiv entgegenzuwirken und soziale Teilhabe zu ermöglichen. Folgerichtig hat der inklusive Breitensport nicht nur hohe Auswirkungen auf die individuellen Entwicklungen von Menschen mit Beeinträchtigung, sondern gleichwohl auch auf die Gesamtgesellschaft hinsichtlich des Abbaus von Ängsten und Unsicherheiten im direkten Umgang mit der Zielgruppe [10].

Somit lässt sich pointieren, dass dem Sport für Menschen mit Beeinträchtigung, auf der Grundlage der UN-BRK, eine besondere Bedeutung in der Praxis zugeschrieben werden kann. „Dem Bereich der gemeinsamen Freizeit kommt hier eine hohe Bedeutung zu, bietet doch gerade das zwanglose Miteinander gute Möglichkeiten, um Vorurteile abzubauen, Freundschaften zu schließen, bestehende Hemmschwellen abzubauen und auf niedrigschwelliger Ebene Inklusion zu leben“ [6].

### Paralympioniken als Vorbilder für Bewegungsförderung und soziale Teilhabe in der Gesellschaft

Im Rahmen einer repräsentativen quantitativen Längsschnittstudie wurde untersucht, welchen Einfluss die Paralympischen Spiele Paris 2024 auf die gesellschaftliche Wahrnehmung sowie die Teilhabe von Menschen mit Beeinträchtigung im Sport haben. Befragt wurden vor, während und nach den Spielen die deutsche Bevölkerung, Menschen mit Beeinträchtigung sowie paralympische Athlet*innen. Die Studie basiert auf standardisierten Online-Befragungen und erfasst insbesondere Einstellungen zu Inklusion, Teilhabe und den Paralympics als Sportgroßereignis [12].

„Die Studie zeigt: Die Paralympics haben einen signifikanten Einfluss auf die gesellschaftliche Wahrnehmung und Inklusion von Menschen mit Beeinträchtigung. Über 60 % der Befragten sind der Meinung, dass die Paralympischen Spiele die Inklusion in der Gesellschaft fördern. Zudem sehen mehr als 70 % der Befragten die Spiele als wichtigen Beitrag gegen Diskriminierung von Menschen mit Beeinträchtigung. Dabei trägt die erhöhte Sichtbarkeit von Para-Sportler*innen laut den Befragten dazu bei, Diskriminierung und stereotype Gesellschaftsbilder abzubauen“ [12].

Die Teilhabe-Community, ein von Ipsos und Aktion Mensch eingerichtetes Befragungspanel zur gezielten Erhebung von Daten unter Menschen mit Beeinträchtigung [13], stimmt zu 83 % der Aussage zu, dass paralympische Athlet*innen als Vorbilder wahrgenommen werden. Die mediale Sichtbarkeit sowie die Verfolgung der leistungssportlichen Erfolge im Kontext der Paralympischen Spiele können dabei als Impulsgeber für Bewegungsförderung innerhalb dieser Zielgruppe verstanden werden. Sie verdeutlichen, dass sportliche Leistungsfähigkeit und aktive Bewegung realisierbar sind, und tragen zur Verfestigung eines positiven und machbaren Selbst- und Gesellschaftsbildes bei.

Hieran anknüpfend zeigt sich in Studien, dass Sport neben seiner gemeinschaftsbildenden Wirkung auch das subjektive Wohlbefinden deutlich positiv beeinflussen kann [10]. Durch körperliche Aktivität werden unter anderem Endorphine ausgeschüttet, die zur Stressreduktion beitragen und die Stimmung verbessern. Darüber hinaus kann regelmäßige Bewegung das Selbstwertgefühl stärken, die psychische Widerstandsfähigkeit (Resilienz) fördern und dazu beitragen, Angstzustände sowie depressive Symptome zu mindern [10]. Unter Berücksichtigung dessen wurden die paralympischen Athlet*innen gefragt, welche Möglichkeiten sozialer Teilhabe durch den Leistungssport entstehen. Die Befragten zeigten insgesamt sehr hohe Zustimmungswerte zum positiven Einfluss des Leistungssports auf soziale Teilhabe und persönliche Ressourcen:

- 77 % geben an, durch Leistungssport besser mit Barrieren im Alltag zurechtzukommen.
- 94 % sehen eine bessere Bewältigung stressiger Lebenssituationen.
- 65 % betrachten die eigene Beeinträchtigung als Stärke.
- 94 % fühlen sich mental widerstandsfähig.
- 94 % berichten, auch in anderen Lebensbereichen eine gute Ausdauer zu haben [12].

Insgesamt zeigt sich damit während der Paralympics eine sehr starke subjektiv empfundene Wirkung des Leistungssports auf Resilienz, Stressbewältigung und Alltagskompetenzen.

## Sportvereine als zentrale Orte der Bewegungsförderung

### Das Spannungsfeld zwischen Teilhabewunsch und realer Sportaktivität

Eghbalpour [3] zeigt in ihrer Dissertation am Beispiel der Stadt Aachen auf, dass lediglich 29,6 % der Menschen mit körperlicher (*N* = 75) und 30,8 % der Menschen mit kognitiver Beeinträchtigung (*N* = 173) aktive Mitglieder in einem breitensportlich ausgerichteten Sportverein sind. Gleichzeitig besteht ein deutlicher Wunsch nach Sportvereinszugehörigkeit: 50,5 % der Befragten äußern ein Interesse, trotz bisheriger sportlicher Inaktivität. Aufseiten der Sportvereine zeigt sich jedoch ein anderes Bild: 89,7 % der Vereinsvorsitzenden (*N* = 58) und 72,3 % der Übungsleitungen (*N* = 130) berichten, dass sie nie oder nur sehr selten Anfragen von Menschen mit Beeinträchtigung erhalten. Dabei besteht grundsätzlich eine hohe Offenheit für die Aufnahme: 48,3 % der Vorsitzenden und 49,2 % der Übungsleitungen geben an, dass Menschen mit Beeinträchtigung an ihren Sportgruppen teilnehmen könnten. Als Handlungsempfehlung für Sportvereine bietet sich die Benennung von Ansprechpartner*innen für Inklusion an. Derzeit haben nur wenige Vereine solche Ansprechpersonen etabliert (22,4 % der Vorsitzenden und 23,1 % der Übungsleitungen). Eine offizielle Ansprechperson könnte nicht nur Kontaktanfragen entgegennehmen und spezifische Rückfragen beantworten, sondern auch aktiv zu einer einladenden und inklusiven Willkommenskultur beitragen.

### Strukturelle Barrieren: Mobilität, Assistenz und Zugänglichkeit

Ferner wird aus den Forschungsergebnissen von Eghbalpour [3] deutlich, dass Menschen mit Beeinträchtigung seltener sportlich aktiv sind als Menschen ohne Beeinträchtigung und zudem nur vereinzelt die Initiative ergreifen, nach konkreten Sportangeboten zu fragen. Eghbalpour [3] gibt dabei interessante Einblicke in den Unterstützungsbedarf im Bereich Mobilität und persönlicher Assistenz im Sport. So bewerten Menschen mit körperlicher Beeinträchtigung (80 %) und kognitiver Beeinträchtigung (89,9 %), die nicht in einem Sportverein aktiv sind, Assistenzleistungen als besonders wichtig. Diese hohe Relevanz wird auch von den befragten sportlich aktiven Menschen mit Beeinträchtigung bestätigt. Eine detaillierte Betrachtung zeigt ebenso, dass insbesondere Menschen mit kognitiver Beeinträchtigung spezifische Unterstützung benötigen: Neben der am häufigsten genannten Unterstützung bei der Durchführung von Sportübungen (aktive Sportler*innen 59,3 %; inaktive 70,6 %) wird der Wunsch nach Fahrdiensten zu den Sportangeboten hervorgehoben (aktive Sportler*innen 61,1 %; inaktive 66,4 %). Trotz dieses klaren Unterstützungsbedarfs zeigt sich, dass entsprechende Leistungen im Bereich der Sportassistenz und Mobilität bisher kaum in Anspruch genommen werden.

Die Studie „Mittendrin statt nur dabei“ verdeutlicht zudem, dass viele Menschen mit Behinderungen auf Unterstützung angewiesen sind, um sportlich aktiv sein zu können. Insbesondere Personen, die in Einrichtungen leben oder allein wohnen, benötigen häufig Assistenzleistungen, um Zugang zu Sport- und Bewegungsangeboten zu erhalten. Dabei spielen sowohl die Verfügbarkeit entsprechender Unterstützungsangebote als auch die Qualifizierung von Fach- und Assistenzkräften eine zentrale Rolle [14].

### Professionalisierung inklusiver Sportpraxis durch Qualifizierung und Haltung

Auf Basis der Forschungsergebnisse von Eghbalpour [3] wird deutlich, dass die Professionalisierung inklusiver Sportpraxis wesentlich durch Qualifizierung und eine reflektierte professionelle Haltung geprägt ist. Insbesondere fehlende Erfahrungswerte und seltene Berührungspunkte im Umgang mit Menschen mit Beeinträchtigung führen in Sportvereinen häufig zu Unsicherheiten. Diese lassen sich durch gezielte Aus- und Fortbildungsangebote im Sport abbauen, die nicht nur fachliches Wissen vermitteln, sondern auch die Entwicklung einer inklusiven Haltung unterstützen. Die Ergebnisse zeigen zudem, dass Qualifizierung als zentraler Bestandteil professioneller Praxis sowohl von Vereinsakteur*innen als auch aus Perspektive der Menschen mit Beeinträchtigung stark eingefordert wird. So bewerten 65,4 % der Übungsleitungen und 86,2 % der Vereinsvorsitzenden entsprechende Qualifizierungsmaßnahmen als entscheidend für eine gelingende Umsetzung inklusiver Sportangebote.

Ein zukunftsweisendes Ziel könnte die Reform der Übungsleiter*innen-C-Lizenz sein, bei der inklusionsspezifische Inhalte in die Ausbildung integriert werden. Mehr als die Hälfte der Übungsleitungen (61,5 %) erachtet eine solche Erweiterung innerhalb der Basisausbildung als sehr wichtig. Derzeit sind inklusionsspezifische Themen in der Grundausbildung kaum vertreten. Die Forschungsergebnisse zeigen jedoch klar auf, welche Lerninhalte in einer reformierten Ausbildung besondere Berücksichtigung finden sollten:

- Erste Hilfe und Notfallversorgung (62,3 %),
- methodisch-didaktisches Fachwissen für inklusive Angebote (60 %),
- Anpassung von Sport‑, Spiel- und Bewegungsangeboten für Menschen mit Beeinträchtigung (61,5 %),
- Rechts- und Versicherungsfragen (51,5 %),
- Ausdauer- und Gerätetraining für Menschen mit Beeinträchtigung (46,9 %),
- Prävention sexualisierter Gewalt im Kontext von Menschen mit Beeinträchtigung (47,7 %).

Trotz des ausgeprägten Bedarfs an Handlungssicherheit durch Qualifizierungsmaßnahmen ist festzuhalten, dass die Umsetzung von Inklusion nicht ausschließlich über Fort- und Weiterbildungen gewährleistet werden kann. Menschen mit Beeinträchtigung nehmen dabei eine zentrale Rolle als Expert*innen in eigener Sache ein. Ihre aktive Beteiligung sowie die selbstbestimmte Artikulation behinderungsbedingter Bedarfe stellen wesentliche Voraussetzungen für eine gelingende Inklusion im Sportverein dar.

## Chancen und Herausforderungen für die sport- und bewegungsorientierte Soziale Arbeit

Im Hinblick auf die Kerngedanken der Bewegungsförderung, stärkeren Teilhabe und Selbstbestimmung stellt sich die Frage, in welcher Weise Menschen mit Beeinträchtigung in einem inklusiven Sozialraum Unterstützung durch die Fachkräfte der Sozialen Arbeit erfahren können, um selbstverständlich Angebots- und Unterstützungsstrukturen im öffentlichen Raum nutzen zu können. Dies hängt u. a. in hohem Maße von der Haltung sowie dem individuellen Verständnis der Fachkräfte in der Sozialen Arbeit ab, welche menschenrechtsorientiert und nicht paternalistisch ausgerichtet sein sollten [15].

Den rechtlichen Verankerungen der UN-BRK folgend, zeigt sich beispielsweise in Artikel 19, dass MmB „Zugang zu einer Reihe von gemeindenahen Unterstützungsdiensten (…), einschließlich der persönlichen Assistenz“, haben. Darüber hinaus schließt sich Artikel 20 der UN-BRK an, welcher den rechtlichen Anspruch auf persönliche Mobilität unterstreicht, verwoben mit Artikel 20a UN-BRK, der die Flexibilität dieser Lösungsmöglichkeit schärft. Auch mit Blick auf das Bundesteilhabegesetz (BTHG), § 78 Abs. 1 Sozialgesetzbuch (SGB) IX wird der Assistenzanspruch im Sport erstmalig festgeschrieben. Nichtsdestotrotz findet dieser keinerlei Berücksichtigung im Teilhabeplanverfahren der MmB. Folgerichtig besteht an dieser Stelle dringender Handlungsbedarf für die Soziale Arbeit, um die aktuelle Situation der MmB bundesweit beleuchten als auch verbessern zu können. „Ein Assistenzbedarf für den Bereich Sport und Bewegung muss nach § 78 SGB IX in der individuellen Teilhabeplanung explizit berücksichtigt sein, damit eine Assistenz beantragt und bewilligt werden kann“ [16]. Zur Verbesserung der Teilhabemöglichkeiten für MmB im Sport bedarf es verständlicher, unbürokratischer und pragmatischer Lösungen für die Beantragung von Assistenzen und Fahrdiensten im Sportvereinssystem. Antragsverfahren müssen sowohl niederschwellig und einheitlich geregelt sein als auch mit deutlich umfassenderen Fachleistungsstunden für Sozialarbeiter*innen im Freizeitbereich versehen werden.

Es lässt sich festhalten, dass es für die Verbesserung von Teilhabemöglichkeiten im Sport erweiterter Handlungs- und Entscheidungsspielräume, Teilhabechancen sowie einer Auswahlmenge an inklusiven Sportangeboten wie auch an Sportangeboten ausschließlich für Menschen mit Beeinträchtigung bedarf. Hierbei liegt der Fokus besonders auf dem Ziel, dass Menschen mit Beeinträchtigung eine Sportaktivität selbstbestimmt und in frei gewählten Kontexten ausüben können. Interdisziplinär sowie im Zuge der UN-BRK betrachtet, bedeutet dies für die praxisnahen Handlungsebenen Folgendes: Es bedarf Aktion und Reaktion, auf der gesamtgesellschaftlichen Ebene, auf der Ebene der politischen Strukturen, auf der Ebene der Sportvereine, der subjektzentrierten Ebene für die Menschen mit Beeinträchtigung. Es bedarf als Ausgangsbasis eines angepassten Verständnisses von Menschen mit Beeinträchtigung in der Gesellschaft sowie eines erweiterten Blickwinkels, der die gesellschaftliche Konstruktion des Behinderungsbegriffs durch Umweltbarrieren hinterfragt und die stark defizitäre Sichtweise verlässt [17]. Gefordert ist folglich ein soziokultureller Wandel in der Gesellschaft mit der Vision von Normalität und uneingeschränkter Teilhabe für Menschen mit Beeinträchtigung im Sport. Hierbei stehen Menschen mit Beeinträchtigung mit ihren sportbezogenen Kompetenzen, Fähigkeiten und Fertigkeiten vor allem als Sportler*innen im Fokus, die ihre Sportangebote frei wählen können. Denn „Teilhabe impliziert Wahlmöglichkeiten“ [18]. Es werden sowohl inklusive Sportangebote als auch Sportangebote, die sich ausschließlich an Menschen mit Beeinträchtigung aus allen Altersklassen richten, gebraucht.

## Fazit

Bewegungsförderung bei Menschen mit Beeinträchtigung ist eng mit Fragen der sozialen Teilhabe, Selbstbestimmung und Chancengleichheit verbunden. Sport- und bewegungsorientierte Soziale Arbeit kann hierbei einen entscheidenden Beitrag leisten, indem sie Menschen mit Beeinträchtigung beim Zugang zu Sportvereinen unterstützt, Barrieren abbaut und inklusive Teilhabestrukturen stärkt.

Die Ausführungen zeigen, dass Sportvereine wichtige Orte gesellschaftlicher Begegnung und Bewegungsförderung sind. Gleichzeitig bestehen weiterhin erhebliche strukturelle Hindernisse, etwa in den Bereichen Mobilität, Assistenz, Barrierefreiheit und Qualifizierung von Übungsleitungen. Dadurch bleibt der Anspruch der UN-Behindertenrechtskonvention auf gleichberechtigte Teilhabe im Sport bislang vielfach unerfüllt.

Gerade hier eröffnen sich zentrale Handlungsfelder für die sport- und bewegungsorientierte Soziale Arbeit. Sie kann Menschen mit Beeinträchtigung bei der Wahrnehmung ihrer Rechte unterstützen, Teilhabeplanungen begleiten, Netzwerke zwischen Vereinen und Unterstützungsstrukturen fördern sowie zu einer inklusiven Haltung im Sport beitragen. Gleichzeitig wird deutlich, dass Inklusion nicht allein durch einzelne Maßnahmen erreicht werden kann, sondern einen gesamtgesellschaftlichen und politischen Wandel erfordert.

Inklusive Bewegungsförderung bedeutet letztlich, Menschen mit Beeinträchtigung selbstbestimmte Wahlmöglichkeiten zur Teilhabe im Sport zu eröffnen und ihre sportlichen Kompetenzen in den Mittelpunkt zu stellen. Erst wenn Sportvereine für alle Menschen selbstverständlich zugänglich sind und Teilhabe unabhängig von Unterstützungsbedarfen ermöglicht wird, kann Inklusion im Sinne der UN-BRK nachhaltig verwirklicht werden.

## Data Availability

Für den vorliegenden Artikel wurden keine Datensätze erstellt oder analysiert. Die analysierten Datensätze sind auf begründete Anfrage bei der Korrespondenzperson erhältlich.

## References

[1] Anneken V (2013) Inklusion durch Sport. Forschung für Menschen mit Behinderungen. Wissenschaftliche Schriftenreihe des Forschungsinstituts für Inklusion durch Bewegung und Sport, Bd 2. Sportverlag Strauß, Köln

[2] Landessportbund Nordrhein-Westfalen (o J) Ehrenamt inklusiv. https://www.lsb.nrw/medien/news/artikel/ehrenamt-inklusiv. Zugegriffen: 16. Juli 2026

[3] Eghbalpour S (2023) Wie können Teilhabemöglichkeiten für Menschen mit Beeinträchtigung im Vereinssport verbessert werden? Ansätze und Ergebnisse einer partizipativen Bedarfsanalyse sowie sportpolitische Handlungsempfehlungen für den organisierten Sport am Beispiel der Stadt Aachen. Deutsche Sporthochschule Köln 10.17883/4786 (Dissertation)

[4] Wansing G (2013) Inklusion und Behinderung. Standortbestimmung und Anfragen an den Sport. In: Volker A (Hrsg) Inklusion durch Sport. Sportverlag Strauß, Köln

[5] Degener T (2018) Die UN-Behindertenrechtskonvention – ein neues Verständnis von Behinderung. In: Degener T, Diehl E (Hrsg) Handbuch der Behindertenrechtskonvention. Bundeszentrale für politische Bildung, Bonn, S 55–65

[6] Kiuppis F, Kurzke-Maasmeier S (Hrsg) (2012) Sport im Spiegel der UN-Behindertenrechtskonvention. Kohlhammer, Stuttgart

[7] Bundesministerium für Arbeit und Soziales (2021) Dritter Teilhabebericht der Bundesregierung über die Lebenslagen von Menschen mit Beeinträchtigungen. BMAS, Bonn

[8] Bundesministerium für Arbeit und Soziales (2025) Repräsentativbefragung zur Teilhabe von Menschen mit Behinderungen (Teilhabesurvey). BMAS, Bonn

[9] Der Paritätische Gesamtverband (2026) Massive Kürzungsvorschläge bei Kindern, Jugendlichen und Menschen mit Behinderung. https://www.der-paritaetische.de/alle-meldungen/massive-kuerzungsvorschlaege-bei-kindern-jugendlichen-und-menschen-mit-behinderung/. Zugegriffen: 3. Mai 2026

[10] Abel T, Anneken V, Schliermann R (2014) Entwicklung des Behindertensports. In: Schliermann R, Anneken V, Abel T, Scheuer T, Froböse I (Hrsg) Sport von Menschen mit Behinderungen: Grundlagen, Zielgruppen, Anwendungsfelder. Urban & Fischer, München, S 2–10

[11] Landessportbund Nordrhein-Westfalen (Hrsg) (2011) Aufbaumodul ÜL-C-Ausbildung. Handreichungen für Teilnehmerinnen und Teilnehmer, 3. Aufl. Schmitzdruck & Media, Grüggen

[12] Aktion Mensch, Katholische Hochschule Nordrhein-Westfalen (2024) Paralympische Spiele und Inklusion: Wie die Paralympics Paris 2024 die gesellschaftliche Wahrnehmung von Menschen mit Beeinträchtigung beeinflussen. https://www.aktion-mensch.de/paralympics-studie. Zugegriffen: 27. Juli 2026 (Forschungsbericht)

[13] Thönnes L (2022) Partizipative Forschung digital und vielfältig. Erprobung eines neuen Formats. Behindertenpädagogik 61(1):57–73

[14] Berufsgenossenschaft für Gesundheitsdienst und Wohlfahrtspflege, Special Olympics Deutschland (2023) Mittendrin statt nur dabei: Zweiter Bericht zum Forschungsprogramm von BGW und SOD zu Teilhabe im Sport. BGW, Hamburg

[15] Wesselmann C (2022) Modelle von Behinderung in den Disability Studies. In: Waldschmidt A (Hrsg) Handbuch Disability Studies. Springer VS, Wiesbaden

[16] Sauerland K, Heitzer J, Tillmann V, Volker A (2018) Assistenz in Bewegung und Sport: Handlungsempfehlung. Forschungsinstitut für Inklusion durch Bewegung und Sport, Frechen

[17] Waldschmidt A (2020) Disability Studies zur Einführung. Junius, Hamburg

[18] Bartelheimer P (2022) Teilhabe – ein Versuch einer Begriffsbestimmung. In: Wansing G, Schäfers M, Köbsell S (Hrsg) Teilhabeforschung – Konturen eines neuen Forschungsfeldes. Springer VS, Wiesbaden, S 13–34

